# Dexamethasone Implant in Patients with Diabetic Macular Edema Resistant to Anti-VEGF Therapy

**DOI:** 10.4274/tjo.galenos.2018.84665

**Published:** 2019-04-30

**Authors:** Serhad Nalçacı, Cezmi Akkın, Filiz Afrashi

**Affiliations:** 1 Ege University Faculty of Medicine, Department of Ophthalmology, İzmir, Turkey

**Keywords:** Dexamethasone, diabetic macular edema, ranibizumab, vascular endothelial growth factor

## Abstract

**Objectives:**

To investigate the efficacy of single dose intravitreal dexamethasone implant in patients with diabetic macular edema (DME) resistant to anti-VEGF therapy.

**Materials and Methods:**

Twenty eyes of 14 patients (8 male, 6 female; mean age, 65±5.7 years) with DME resistant to intravitreal ranibizumab injections were studied. A single intravitreal dexamethasone implant was injected into each eye and patients were followed up for 6 months. Response to therapy was assessed monthly by measuring intraocular pressure (IOP), best-corrected visual acuity (BCVA), and central foveal thickness (CFT).

**Results:**

Baseline (before injection) IOP was 14.9±2.7 mmHg and did not change significantly in the six months following injection. Baseline BCVA was 1.04±0.35 LogMAR and improved to 0.86±0.31 at month 1 without statistical significance (p=0.056). CFT was significantly lower in all monthly measurements compared to its baseline value of 682.2±229.2 μm. During the follow-up period, endophthalmitis, significant cataract, or rhegmatogenous retinal detachment were not detected.

**Conclusion:**

Intravitreal dexamethasone implant injection is associated with significant CFT reduction for up to six months without causing any complications. Although BCVA did not improve in parallel with the CFT reduction, intravitreal dexamethasone implant should be considered as an effective and safe treatment option in the management of DME patients resistant to anti-VEGF injections.

## Introduction

Diabetic macular edema (DME) is the leading cause of visual loss in patients with diabetes, affecting an estimated 21 million individuals worldwide.^1^ The presence of DME varies with the duration and stage of diabetic retinopathy. Its prevalence is 3% in mild non-proliferative retinopathy, 38% in moderate to severe non-proliferative retinopathy, and 71% in eyes with proliferative retinopathy.^2^ Almost 50% of patients with DME lose two or more lines of visual acuity within two years of diagnosis.^3^

Macular laser photocoagulation has long been the standard of care of DME because it is effective in preserving vision.^4^ However, this procedure has a limited effect in restoring lost vision, which may be due to expanded retinal scars over time and decrease in vision and contrast sensitivity.^5^ The efficacy of intravitreal injection of vascular endothelial growth factor inhibitors (anti-VEGF) has been proven in several randomized clinical trials, which reported better outcomes compared to macular laser photocoagulation in DME.^6,7^ However, not all patients respond favorably to intravitreal anti-VEGF therapy.

Although first-line treatment of DME with anti-VEGF agents has become the gold standard, there is no consensus on the treatment of patients who do not respond to anti-VEGF agents.^8^ Dexamethasone, a potent corticosteroid, has been approved for various ocular pathologies including DME and is recommended particularly in refractory cases.^9^ Although many recent studies focused on the effect of intravitreal dexamethasone implant in DME patients resistant to anti-VEGF therapy, more evidence is still needed to draw more detailed conclusions.^7,10,11^ Therefore, we aimed to investigate the efficacy of a single intravitreal dexamethasone implant in patients with DME that did not respond to multiple ranibizumab injections.

## Materials and Methods

### Study Design and Population

This was a retrospective study. Patients over 18 years of age who had DME resistant to at least 3 monthly ranibizumab injections and central foveal thickness (CFT) ≥300 µm on spectral-domain optical coherence tomography (SD-OCT) (Topcon 3D OCT-2000) were included in the study. Exclusion criteria were macular ischemia on fluorescein angiography (FA), retinal vasculopathies other than diabetic retinopathy, vitreomacular traction, glaucoma, ocular hypertension, advanced cataract, uncontrolled systemic disease, history of vitreoretinal surgery, intraocular surgery within the last 6 months, panretinal or focal laser treatments within the last 3 months, and being a steroid responder.

The study was conducted in compliance with Declaration of Helsinki and was approved by Ethics Committee of the Ege University Faculty of Medicine. Written informed consent was obtained from all patients enrolled.

### Intravitreal Dexamethasone Implant Injection

After eye cleansing with 5% povidone-iodine solution, an intravitreal dexamethasone implant (Ozurdex^®^, Allergan Inc., Irvine, CA, USA) was injected through the pars plana under topical anesthesia in sterile operating room conditions. Topical ophthalmic antibiotic was applied for 7 days after the injection. After the injection, the patients were assessed monthly during a 6-month follow-up period.

### Study Parameters

FA was performed only at the initial visit. Evaluation by slit-lamp biomicroscopy and fundus examination were performed at every visit. Response to the intravitreal dexamethasone implant was assessed monthly by measuring intraocular pressure (IOP) in mmHg, best-corrected visual acuity (BCVA) in logarithm of the minimum angle of resolution (LogMAR), and CFT measured on SD-OCT in µm. If IOP was over 25 mmHg, a topical anti-glaucoma medication was started.

### Statistical Analysis

Study data were summarized using descriptive statistics such as mean, standard deviation, range, frequency, and percentage. Repeated measures analysis of variance (ANOVA) was used to test the statistical significance of change in continuous variables over time. The limit for statistical significance was set as p<0.05. All statistical analyses were performed with SPSS for Windows (Statistical Package for Social Sciences, ver. 22.0, SPSS Inc., Chicago, IL, USA) software.

## Results

Twenty eyes of 14 patients (8 male, 6 female; mean age, 65±5.7 years) were included in the study. Half of these eyes were phakic and other half were pseudophakic. The eyes had not responded to an average of 4.85 (range, 3-10) ranibizumab injections. Panretinal or focal laser treatments had previously been applied to all eyes (Table 1).

**Figure 1:**
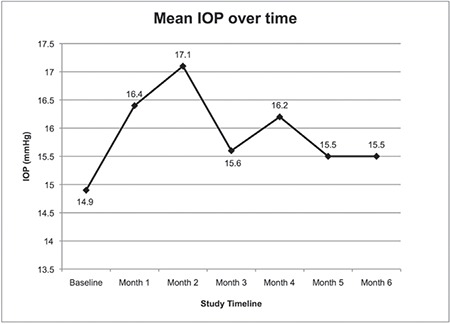
Mean intraocular pressure before (baseline) and over six months after the intravitreal dexamethasone implant injection IOP: Intraocular pressure

**Figure 2:**
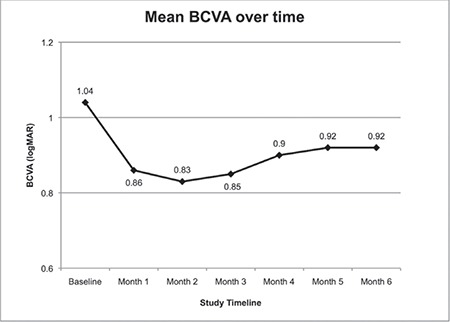
Mean best-corrected visual acuity before (baseline) and over six months after the intravitreal dexamethasone implant injection (LogMAR, logarithm of the minimum angle of resolution) BCVA: Best-corrected visual acuity

**Figure 3:**
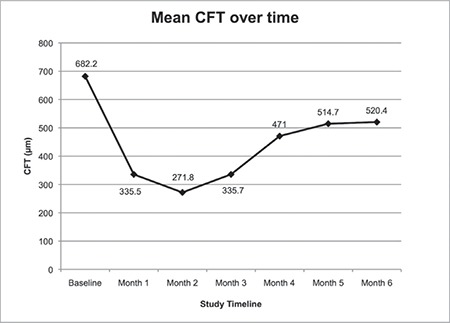
Mean central foveal thickness before (baseline) and over six months after the intravitreal dexamethasone implant injection CFT: Central foveal thickness

Intravitreal dexamethasone implant injection was performed in both eyes of 6 patients (42.8%). In 5 eyes (25%), IOP increased to 25-30 mmHg within 1-3 months after the procedure, which was successfully treated with topical anti-glaucoma medication. Baseline (before injection) IOP was 14.9±2.7 mmHg and did not change significantly in the 6 months following injection (Table 2, Figure 1). Baseline BCVA was 1.04±0.35 LogMAR and improved to 0.86±0.31 LogMAR at 1 month after injection but without statistical significance (p=0.056). It remained at about this level over 6 months of follow-up with no additional improvement (Table 2, Figure 2). On the other hand, CFT was significantly lower in all monthly measurements after implant injection compared to its baseline value of 682.2±229.2 µm (Table 2, Figure 3). During the 6-month follow-up period, none of the eyes developed endophthalmitis, significant cataract, or rhegmatogenous retinal detachment.

## Discussion

In this retrospective study, we evaluated the efficacy of a single intravitreal dexamethasone implant injection in patients with persistent DME after at least three ranibizumab injections. We primarily found that intravitreal dexamethasone implant injection effectively reduced CFT starting at 1 month and lasting up to 6 months after injection. The BCVA was also improved with intravitreal dexamethasone implant injection, but this improvement did not reach a statistical significance. Nevertheless, our findings indicate that intravitreal dexamethasone implant can be considered in patients with DME resistant to anti-VEGF therapy.

Today, the treatment paradigm for DME has shifted away from laser and toward intravitreal pharmacotherapy particularly with anti-VEGF agents.^8^ Although treatment with anti-VEGF injections has obviously proved its efficacy, some patients exhibit poor functional and anatomic response.^12^ Furthermore, multiple anti-VEGF injections can result in increased morbidity, multiple hospital visits, and severe damage to photoreceptors and retinal pigment epithelium in chronic DME.^7^ It has also been shown that visual prognosis for many macular diseases is also correlated with integrity of the photoreceptor inner and outer segments junction and external limiting membrane lines on spectral domain OCT.^13,14^ Therefore, intravitreal injection of corticosteroids has been suggested for the treatment of DME patients resistant to anti-VEGF therapy and to limit the number of injections for better visual prognosis.^8^

Dexamethasone is presented in the form of an intravitreal sustained-release implant, which has the advantage of extending the duration of intravitreal activity and limiting the number of injections.^9^ The dexamethasone intravitreal implant showed similar or better outcomes for vision-related quality of life, CMT reduction, and BCVA improvement compared to anti-VEGF therapy in comparative studies.^15,16,17,18^ Even a simultaneous treatment regimen combining two drugs has been suggested to provide effective management of DME with an acceptable safety profile.^19^

In recent studies, intravitreal dexamethasone implant in DME patients resistant to anti-VEGF therapy has been shown to improve both BCVA and CMT in both the short term and long term up to 18 months.^20,21,22,23^ In a very recent meta-analysis of 3859 patients from 15 studies, intravitreal dexamethasone implant was found to be associated with significant mean improvement in BCVA in patients with DME who have a suboptimal response to anti-VEGF therapy.^24^ In accordance with this literature, we found that dexamethasone implant was effective in patients with persistent DME. It decreased CFT from 1 to 6 months after the injection. The peak efficacy of the implant was reached at 1-3 months, then it decreased in months 4-6. Totan et al.^23^ also reported that the therapeutic efficacy of intravitreal dexamethasone implant decreases between 3 and 6 months after injection. In contrast to the literature, in our study BCVA did not improve in parallel with the CFT decrement.^25,26^ Visual prognosis for many macular diseases is correlated with integrity of the photoreceptor inner and outer segment junction and external limiting membrane lines on spectral domain OCT.^13,14^ Chronic DME can also result in damage to photoreceptors and the retina pigment epithelium.^7^ Therefore, avoiding delay in treatment of DME can result in better visual outcomes. In addition, the dexamethasone implant may facilitate longer sustained control of DME.

The incidence of IOP elevation was 25% in our study, which was similar to the 13-30% in the previous reports.^27,28,29^ In addition, mean IOP did not show significant change during the study. In contrast to some previous studies that a reported high rate of cataract formation associated with intravitreal dexamethasone after multiple injections,^11,15,30^ advanced cataract formation was not observed in our series, indicating that steroid-induced cataract is not a limiting factor for a single intravitreal dexamethasone implant.

### Study Limitations

The main limitation of our study was its small sample size, which limits statistical power and precludes us from reaching a definitive conclusion. Nevertheless, our findings contribute to the literature on the use of intravitreal dexamethasone implant in the management of DME patients resistant to regular anti-VEGF injections.

## Conclusion

Intravitreal dexamethasone implant injection is associated with significant CFT reduction for up to six months without causing any complications. Although our results did not show improvement in BCVA in parallel with the CFT reduction, intravitreal dexamethasone implant should still be considered as an effective and safe treatment option in the management of DME patients resistant to regular anti-VEGF injections.

## Figures and Tables

**Table 1:**
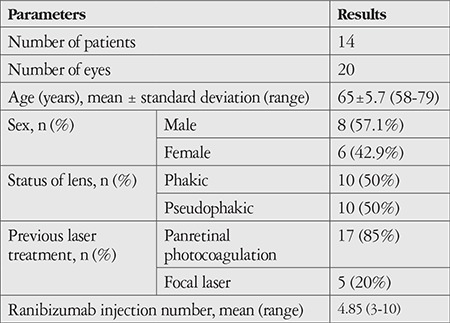
Baseline demographics and ophthalmological history of patients

**Table 2:**
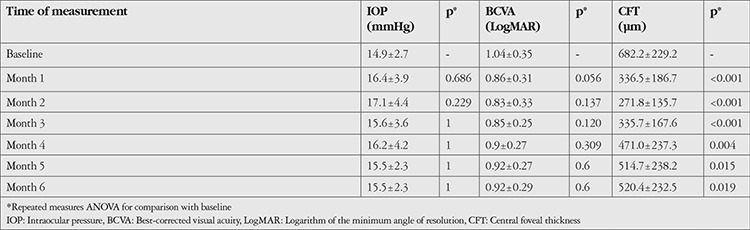
Outcome of ophthalmological evaluation during six months after intravitreal dexamethasone implant injection
